# Supplementary material for: Factors associated with drinking behaviour during COVID-19 social distancing and lockdown among adults in the UK
Source: Drug Alcohol Depend. 2021 Feb 1;219:108461. doi: 10.1016/j.drugalcdep.2020.108461 (PMC7807168; doi:10.1016/j.drugalcdep.2020.108461)
Supplement: Supplementary file 1 [file mmc1.docx]

# Supplementary File 1

## Measures

### Changes in drinking over the past week

Changes in drinking over the past week during social distancing and lockdown were measured using the following question: “Over the past week have you drunk alcohol more than usual?” with response options: a) less than usual; b) about the same; c) more than usual; and d) I don’t drink alcohol. Only drinkers (i.e. those who responded a, b or c) were included in the analysis of changes in drinking over the past week. For analysis of drinking less than usual, drinkers who responded ‘a’ were coded 1 and those who responded ‘b’ were coded 0. For analysis of drinking more than usual, drinkers who responded ‘c’ were coded 1 and those who responded ‘b’ were coded 0.

### Heaviness of drinking in the past week

Heaviness of drinking in the past week was assessed using the question: “How many alcoholic drinks have you had in the past week (e.g. how many glasses of wine / pints of beer or cider / shots of spirits)?” with continuous response options from ‘0’ through to ‘20’ and then ‘21+’. These response options were coded from ‘0’ through to ‘21’ for the primary analyses with sensitivity analyses excluding the response option ‘21+’ because it is non-linear.

### Sociodemographic characteristics

The following sociodemographic characteristics were included: age (continuous in years), sex (% female), ethnicity (% white), level of education (% with post-16 qualifications), and annual household income (% >£30,000 [16]). Level of education and income were used as measures of socioeconomic position (SEP). Level of education provides a reliable indication of SEP prior to COVID-19 as it is not affected by recent job loss or furlough and previous research has also shown that it is one of the strongest socioeconomic predictors of volume of alcohol consumption in England [17]. Income gives a strong indication of the economic resources available to the participant, and has a dose-response association with health and can influence a wide range of circumstances with direct implications for health [18].

### Diagnosed or suspected COVID-19

Participants were asked: “Have you had COVID-19 (coronavirus)?” with response options a) yes diagnosed and recovered, b) yes diagnosed and still ill, c) not formally diagnosed but suspected, d) no. Diagnosed or suspected COVID-19 was coded 1 for those who responded ‘a’, ‘b’ or ‘c’ and 0 for those who responded ‘d’.

### Adherence to COVID-19 protective behaviours

Adherence to COVID-19 protective behaviours was assessed with the question: “Are you following the recommendation from authorities to prevent spread of COVID-19?” with responses on a scale from 1 (not at all) to 7 (very much so). Responses of 5 and above were coded 1 (indicating adherence) and responses of 4 and below were coded 0.

### Stress about COVID-19, finances or boredom

Stress about COVID-19, finances or boredom was assessed with the question: “Have any of these things been causing you significant stress? (e.g. they have been constantly on your mind or have been keeping you awake at night)”. Response options included i) catching COVID-19, ii) becoming seriously ill from COVID-19, iii) finances, and iv) boredom. For each of these four response options, a variable was created where those who reported stress about the relevant outcome were coded 1, else they were coded 0.

### Recent drop in household income

Recent drop in household income was assessed with the question “Have you experienced any of the following in the past week?”. Those who reported ‘lost your job/been unable to do paid work’, ‘your spouse/partner lost their job or was unable to do paid work’ or ‘major cut in household income (e.g. due to you or your partner being furloughed/put on leave/not receiving sufficient work)’ were coded 1 for recent drop in household income and those who reported none of these were coded 0.

### Key worker status

Key worker status may be associated with increased exposure to COVID-19. Participants were asked: “Are you currently fulfilling any of the government’s identified ‘key worker’ roles?” Participants were coded 1 if they responded ‘health, social care or relevant related support work’, ‘teacher or childcare worker still travelling in to work’, ‘transport worker still travelling in to work’, ‘food chain worker (e.g. production, sale, delivery)’, ‘key public services worker (e.g. justice staff, religious staff, public service journalist or mortuary worker)’, ‘local or national government worker delivering essential public services’, ‘utility worker (e.g. energy, sewerage, postal service)’, ‘public safety or national security worker’, or ‘worker involved in medicines or protective equipment production or distribution’. Those who responded ‘none of these’ were coded 0.

### Health conditions

The presence of health conditions was assessed with the question: “Do you have any of the following medical conditions?” Those who selected ‘high blood pressure’, ‘diabetes’, ‘heart disease’, ‘lung disease (e.g. asthma or COPD)’, or ‘cancer’ were coded 1 and those who selected none of these were coded 0. The presence of anxiety disorders was assessed with the same question, with those who selected ‘clinically-diagnosed anxiety’ coded 1 and those who did not select this response coded 0.

### Survey date

Survey date was coded 0 for those surveys completed on 21^st^, 22^nd^ or 23^rd^ March (i.e. prior to lockdown commencing), and subsequent dates were coded 1, 2, 3,…, n (i.e. reflecting the progression of time from the start of lockdown).
