# Supplementary material for: Factors associated with drinking behaviour during COVID-19 social distancing and lockdown among adults in the UK
Source: Drug Alcohol Depend. 2021 Feb 1;219:108461. doi: 10.1016/j.drugalcdep.2020.108461 (PMC7807168; doi:10.1016/j.drugalcdep.2020.108461)
Supplement: Supplementary file 2 [file mmc2.docx]

# Supplementary Tables

Supplementary Table 1: Participant characteristics (unweighted)

|  | Whole sample  % (n), unless otherwise stated |
| --- | --- |
| Age, mean years (SD) | 48.0 (14.52) |
| Female | 74.5 (22,625) |
| White ethnicity | 94.3 (28,646) |
| Post-16 education qualifications | 86.7 (26,335) |
| Annual household income > £30,000 | 63.5 (19,282) |
| Diagnosed or suspected COVID-19 | 10.0 (3,025) |
| Adherence to COVID-19 protective behaviours | 97.6 (29,648) |
| Significant stress about catching COVID-19 | 17.5 (5,318) |
| Significant stress about becoming seriously ill from COVID-19 | 24.4 (7,420) |
| Significant stress about finances | 17.1 (5,196) |
| Significant stress about boredom | 4.3 (1,308) |
| Recent drop in household income | 6.5 (1,969) |
| Key worker | 25.7 (7,812) |
| Physical health conditions^a^ | 27.9 (8,471) |
| Diagnosed anxiety disorder | 12.3 (3,732) |

^a^ Physical health conditions = high blood pressure, diabetes, heart or lung disease, or cancer

Supplementary Table 2: Factors associated with drinking less than usual, compared with same as usual, over the past week (unweighted)

|  | Proportion drinking less than usual, % (n) | Unadjusted |  | Adjusted^b^ |  |
| --- | --- | --- | --- | --- | --- |
|  |  | OR (95% CI) | p | OR_adj_ (95% CI) | p |
| Age, in years | - | 0.88 (0.85, 0.91) | <.001 | 0.86 (0.82, 0.89) | <.001 |
| Sex: Male | 34.5 (1,603) |  |  |  |  |
| Female | 29.8 (3,164) | 0.81 (0.75, 0.87) | <.001 | 0.73 (0.67, 0.79) | <.001 |
| Ethnicity: Ethnic minority | 41.5 (309) |  |  |  |  |
| White | 30.7 (4,458) | 0.62 (0.54, 0.72) | <.001 | 0.68 (0.59, 0.80) | <.001 |
| Post-16 education qualifications: No | 34.4 (654) |  |  |  |  |
| Yes | 30.8 (4,113) | 0.85 (0.77, 0.94) | .002 | 0.86 (0.77, 0.96) | .007 |
| Annual household income: < £30,000 | 33.8 (1,784) |  |  |  |  |
| > £30,000 | 29.9 (2,983) | 0.83 (0.78, 0.90) | <.001 | 0.80 (0.74, 0.87) | <.001 |
| Diagnosed or suspected COVID-19: No | 29.4 (4,022) |  |  |  |  |
| Yes | 47.2 (745) | 2.15 (1.93, 2.39) | <.001 | 2.09 (1.88, 2.33) | <.001 |
| Adherence to COVID-19 protective behaviours: No | 27.6 (97) |  |  |  |  |
| Yes | 31.3 (4,670) | 1.19 (0.95, 1.52) | .142 | 1.31 (1.03, 1.68) | .029 |
| Significant stress about catching COVID-19: No | 30.5 (3,988) |  |  |  |  |
| Yes | 35.7 (779) | 1.27 (1.15, 1.39) | <.001 | 1.03 (0.92, 1.16) | .633 |
| Significant stress about becoming seriously ill from COVID-19: No | 29.7 (3,594) |  |  |  |  |
| Yes | 37.3 (1,173) | 1.41 (1.30, 1.53) | <.001 | 1.31 (1.19, 1.45) | <.001 |
| Significant stress about finances: No | 30.5 (4,014) |  |  |  |  |
| Yes | 35.6 (753) | 1.26 (1.14, 1.38) | <.001 | 1.02 (0.92, 1.13) | .664 |
| Significant stress about boredom: No | 30.8 (4,538) |  |  |  |  |
| Yes | 41.9 (229) | 1.62 (1.36, 1.92) | <.001 | 1.33 (1.11, 1.59) | .002 |
| Recent drop in household income: No | 30.8 (4,438) |  |  |  |  |
| Yes | 37.5 (329) | 1.35 (1.17, 1.55) | <.001 | 1.20 (1.03, 1.40) | .019 |
| Key worker: No | 31.2 (3,591) |  |  |  |  |
| Yes | 31.2 (1,176) | 1.00 (0.92, 1.08) | .999 | 0.94 (0.87, 1.03) | .176 |
| Physical health conditions^a^: No | 30.7 (3,396) |  |  |  |  |
| Yes | 32.7 (1,371) | 1.10 (1.02, 1.19) | .014 | 1.14 (1.05, 1.24) | .002 |
| Diagnosed anxiety disorder: No | 30.7 (4,254) |  |  |  |  |
| Yes | 36.6 (513) | 1.31 (1.16, 1.46) | <.001 | 1.09 (0.96, 1.23) | .165 |
| Survey date | - | 1.01 (1.00, 1.03) | .021 | 1.01 (1.00, 1.02) | .181 |

^a^ Physical health conditions = high blood pressure, diabetes, heart or lung disease, or cancer.
^b^ Multivariable model fully adjusted for all variables in the table.

Supplementary Table 3: Factors associated with drinking more than usual, compared with same as usual, over the past week (unweighted)

|  | Proportion drinking more than usual, % (n) | Unadjusted |  | Adjusted^b^ |  |
| --- | --- | --- | --- | --- | --- |
|  |  | OR (95% CI) | p | OR_adj_ (95% CI) | p |
| Age, in years | - | 0.66 (0.64, 0.68) | <.001 | 0.73 (0.70, 0.75) | <.001 |
| Sex: Male | 31.6 (1,405) |  |  |  |  |
| Female | 42.2 (5,442) | 1.58 (1.47, 1.70) | <.001 | 1.38 (1.28, 1.49) | <.001 |
| Ethnicity: Ethnic minority | 40.4 (295) |  |  |  |  |
| White | 39.4 (6,552) | 0.96 (0.83, 1.12) | .596 | 1.15 (0.99, 1.35) | .071 |
| Post-16 education qualifications: No | 30.2 (541) |  |  |  |  |
| Yes | 40.5 (6,306) | 1.57 (1.42, 1.75) | <.001 | 1.27 (1.13, 1.42) | <.001 |
| Annual household income: < £30,000 | 32.2 (1,660) |  |  |  |  |
| > £30,000 | 42.5 (5,187) | 1.56 (1.45, 1.67) | <.001 | 1.43 (1.33, 1.54) | <.001 |
| Diagnosed or suspected COVID-19: No | 39.0 (6,176) |  |  |  |  |
| Yes | 44.6 (671) | 1.26 (1.13, 1.40) | <.001 | 1.05 (0.94, 1.18) | .354 |
| Adherence to COVID-19 protective behaviours: No | 38.9 (162) |  |  |  |  |
| Yes | 39.5 (6,685) | 1.02 (0.84, 1.25) | .224 | 0.93 (0.75, 1.14) | .483 |
| Significant stress about catching COVID-19: No | 38.2 (5,630) |  |  |  |  |
| Yes | 46.4 (1,217) | 1.40 (1.29, 1.52) | <.001 | 1.11 (1.00, 1.23) | .041 |
| Significant stress about becoming seriously ill from COVID-19: No | 37.4 (5,086) |  |  |  |  |
| Yes | 47.2 (1,761) | 1.50 (1.39, 1.61) | <.001 | 1.33 (1.22, 1.46) | <.001 |
| Significant stress about finances: No | 37.8 (5,555) |  |  |  |  |
| Yes | 48.7 (1,292) | 1.56 (1.43, 1.69) | <.001 | 1.39 (1.27, 1.52) | <.001 |
| Significant stress about boredom: No | 39.1 (6,544) |  |  |  |  |
| Yes | 48.8 (303) | 1.48 (1.26, 1.74) | <.001 | 1.21 (1.02, 1.43) | .029 |
| Recent drop in household income: No | 38.7 (6,277) |  |  |  |  |
| Yes | 51.0 (570) | 1.65 (1.46, 1.86) | <.001 | 1.24 (1.08, 1.41) | .002 |
| Key worker: No | 38.2 (4,881) |  |  |  |  |
| Yes | 43.2 (1,966) | 1.23 (1.15, 1.32) | <.001 | 1.04 (0.97, 1.12) | .274 |
| Physical health conditions^a^: No | 41.2 (5,383) |  |  |  |  |
| Yes | 34.2 (1,464) | 0.74 (0.69, 0.80) | <.001 | 0.94 (0.87, 1.02) | .158 |
| Diagnosed anxiety disorder: No | 38.4 (5,985) |  |  |  |  |
| Yes | 49.3 (862) | 1.56 (1.41, 1.72) | <.001 | 1.23 (1.11, 1.36) | <.001 |
| Survey date | - | 1.05 (1.04, 1.06) | <.001 | 1.04 (1.03, 1.06) | <.001 |

^a^ Physical health conditions = high blood pressure, diabetes, heart or lung disease, or cancer.
^b^ Multivariable model fully adjusted for all variables in the table.

Supplementary Table 4: Sensitivity analysis for factors associated with heaviness of drinking excluding the non-linear response option ‘21+’

|  | Mean number of drinks in past week (SD) | Unadjusted |  | Adjusted^b^ |  |
| --- | --- | --- | --- | --- | --- |
|  |  | B (95% CI) | p | B_adj_ (95% CI) | p |
| Age, in years | - | 0.46 (0.38, 0.54) | <.001 | .52 (0.44, 0.61) | <.001 |
| Sex: Male | 4.1 (4.02) |  |  |  |  |
| Female | 2.9 (4.98) | -1.18 (-1.34, -1.02) | <.001 | -0.92 (-1.08, -0.76) | <.001 |
| Ethnicity: Ethnic minority | 2.3 (3.74) |  |  |  |  |
| White | 3.7 (4.63) | 1.40 (1.12, 1.69) | <.001 | 1.26 (0.97, 1.55) | <.001 |
| Post-16 education qualifications: No | 3.1 (4.49) |  |  |  |  |
| Yes | 3.7 (4.57) | 0.58 (0.39, 0.78) | <.001 | 0.51 (0.31, 0.72) | <.001 |
| Annual household income: < £30,000 | 2.8 (4.26) |  |  |  |  |
| > £30,000 | 4.1 (4.72) | 1.37 (1.20, 1.53) | <.001 | 1.36 (1.19, 1.54) | <.001 |
| Diagnosed or suspected COVID-19: No | 3.5 (4.56) |  |  |  |  |
| Yes | 3.3 (4.51) | -0.15 (-0.44, 0.13) | .285 | 0.03 (-0.25, 0.31) | .824 |
| Adherence to COVID-19 protective behaviours: No | 3.5 (5.03) |  |  |  |  |
| Yes | 3.5 (4.54) | 0.03 (-0.54, 0.61) | .911 | -0.29 (-0.84, 0.27) | .312 |
| Significant stress about catching COVID-19: No | 3.7 (4.64) |  |  |  |  |
| Yes | 2.7 (4.11) | -0.96 (-1.15, -0.77) | <.001 | -0.29 (-0.52, -0.06) | .015 |
| Significant stress about becoming seriously ill from COVID-19: No | 3.7 (4.67) |  |  |  |  |
| Yes | 2.8 (4.09) | -0.92 (-1.09, -0.75) | <.001 | -0.30 (-0.50, -0.10) | .004 |
| Significant stress about finances: No | 3.6 (4.58) |  |  |  |  |
| Yes | 2.9 (4.42) | -0.73 (-0.94, -0.52) | <.001 | 0.005 (-0.21, 0.22) | .966 |
| Significant stress about boredom: No | 3.5 (4.57) |  |  |  |  |
| Yes | 2.9 (4.24) | -0.61 (-0.96, -0.25) | <.001 | 0.03 (-0.31, 0.37) | .853 |
| Recent drop in household income: No | 3.5 (4.55) |  |  |  |  |
| Yes | 3.5 (4.68) | 0.04 (-0.32, 0.40) | .844 | 0.39 (0.02, 0.76) | .040 |
| Key worker: No | 3.6 (4.66) |  |  |  |  |
| Yes | 3.2 (4.20) | -0.30 (-0.48, -0.13) | <.001 | -0.21 (-0.38, -0.04) | .017 |
| Physical health conditions^a^: No | 3.5 (4.46) |  |  |  |  |
| Yes | 3.4 (4.75) | -0.15 (-0.34, 0.03) | .104 | -0.35 (-0.54, -0.15) | <.001 |
| Diagnosed anxiety disorder: No | 3.7 (4.64) |  |  |  |  |
| Yes | 2.2 (3.73) | -1.46 (-1.65, -1.27) | <.001 | -0.68 (-0.87, -0.48) | <.001 |
| Survey date | - | 0.01 (-0.02, 0.04) | .603 | 0.02 (-0.01, 0.05) | .144 |

^a^ Physical health conditions = high blood pressure, diabetes, heart or lung disease, or cancer.
^b^ Multivariable model fully adjusted for all variables in the table.

Note: All data are weighted to match the UK adult population on sex, age, ethnicity, education, and country of living.

Supplementary Table 5: Factors associated with heaviness of drinking in the past week (unweighted)

|  | Mean number of drinks in past week (SD) | Unadjusted |  | Adjusted^b^ |  |
| --- | --- | --- | --- | --- | --- |
|  |  | B (95% CI) | p | B_adj_ (95% CI) | p |
| Age, in years | - | 0.58 (0.52, 0.64) | <.001 | .68 (0.62, 0.74) | <.001 |
| Sex: Male | 5.3 (6.01) |  |  |  |  |
| Female | 3.7 (4.81) | -1.56 (-1.70, -1.43) | <.001 | -1.26 (-1.40, -1.13) | <.001 |
| Ethnicity: Ethnic minority | 2.3 (3.86) |  |  |  |  |
| White | 4.2 (5.24) | 1.89 (1.64, 2.14) | <.001 | 1.71 (1.47, 1.96) | <.001 |
| Post-16 education qualifications: No | 3.3 (5.04) |  |  |  |  |
| Yes | 4.3 (5.20) | 0.95 (0.78, 1.12) | <.001 | 0.77 (0.60, 0.95) | <.001 |
| Annual household income: < £30,000 | 3.2 (4.91) |  |  |  |  |
| > £30,000 | 4.6 (5.27) | 1.41 (1.29, 1.53) | <.001 | 1.48 (1.35, 1.61) | <.001 |
| Diagnosed or suspected COVID-19: No | 4.2 (5.23) |  |  |  |  |
| Yes | 3.7 (4.76) | -0.50 (-0.69, -0.30) | <.001 | -0.31 (-0.50, -0.12) | .002 |
| Adherence to COVID-19 protective behaviours: No | 5.0 (6.28) |  |  |  |  |
| Yes | 4.1 (5.16) | -0.87 (-1.25, -0.49) | <.001 | -1.03 (-1.41, -0.66) | <.001 |
| Significant stress about catching COVID-19: No | 4.3 (5.23) |  |  |  |  |
| Yes | 3.4 (4.91) | -0.88 (-1.03, -0.72) | <.001 | -0.25 (-0.44, -0.07) | .006 |
| Significant stress about becoming seriously ill from COVID-19: No | 4.3 (5.26) |  |  |  |  |
| Yes | 3.5 (4.91) | -0.84 (-0.98, -0.70) | <.001 | -0.26 (-0.42, -0.10) | .001 |
| Significant stress about finances: No | 4.2 (5.20) |  |  |  |  |
| Yes | 3.5 (5.09) | -0.70 (-0.86, -0.55) | <.001 | -0.03 (-0.18, 0.13) | .753 |
| Significant stress about boredom: No | 4.1 (5.18) |  |  |  |  |
| Yes | 3.8 (5.38) | -0.36 (-0.65, -0.07) | .015 | 0.25 (-0.04, 0.53) | .087 |
| Recent drop in household income: No | 4.1 (5.17) |  |  |  |  |
| Yes | 4.3 (5.38) | 0.13 (-0.11, 0.37) | .277 | 0.37 (0.13, 0.62) | .003 |
| Key worker: No | 4.2 (5.30) |  |  |  |  |
| Yes | 3.8 (4.84) | -0.46 (-0.60, -0.33) | <.001 | -0.31 (-0.44, -0.17) | <.001 |
| Physical health conditions^a^: No | 4.2 (5.09) |  |  |  |  |
| Yes | 4.0 (5.42) | -0.24 (-0.37,-0.11) | <.001 | -0.48 (-0.62, -0.35) | <.001 |
| Diagnosed anxiety disorder: No | 4.3 (5.20) |  |  |  |  |
| Yes | 3.1 (4.95) | -1.17 (-1.34, -0.99) | <.001 | -0.35 (-0.53, -0.17) | <.001 |
| Survey date | - | 0.04 (0.02, 0.06) | <.001 | 0.04 (0.02, 0.06) | <.001 |

^a^ Physical health conditions = high blood pressure, diabetes, heart or lung disease, or cancer.
^b^ Multivariable model fully adjusted for all variables in the table.
